# Intraindividual cognitive variability predicts amyloid beta, tau PET, and dementia conversion in Down syndrome: a potential marker of cognitive resilience

**DOI:** 10.1002/alz.71537

**Published:** 2026-06-06

**Authors:** Luciana Mascarenhas Fonseca, Eardi Lila, Ali Shojaie, Jasmeer Chhatwal, Orestes Forlenza, Michal Schnaider Beeri, Sharon Krinsky‐McHale, Ann D. Cohen, Bradley T. Christian, Elizabeth Head, Mark Mapstone, Benjamin Handen, Beau M. Ances, Thomas Grabowski, Naomi S. Chaytor, Shahid Zaman, for the Alzheimer's Biomarkers Consortium‐ Down Syndrome (ABC‐DS) Investigators

**Affiliations:** ^1^ The Krieger Klein Alzheimer's Research Center Rutgers Health New Brunswick New Jersey USA; ^2^ Programa Terceira Idade (PROTER, Old Age Research Group), Department and Institute of Psychiatry University of São Paulo School of Medicine São Paulo São Paulo Brazil; ^3^ Department of Biostatistics University of Washington Seattle Washington USA; ^4^ Department of Neurology Massachusetts General Hospital and Harvard Medical School Boston Massachusetts USA; ^5^ Brigham and Women's Hospital Boston Massachusetts USA; ^6^ Laboratory of Neuroscience (LIM‐27), Department and Institute of Psychiatry University of São Paulo School of Medicine São Paulo São Paulo Brazil; ^7^ New York State Institute for Basic Research in Developmental Disabilities New York New York USA; ^8^ Department of Psychiatry University of Pittsburgh Pittsburgh Pennsylvania USA; ^9^ Wisconsin Alzheimer's Disease Research Center, School of Medicine and Public Health University of Wisconsin‐Madison Madison Wisconsin USA; ^10^ Department of Pathology and Laboratory Medicine University of California Irvine California USA; ^11^ Department of Neurology, Institute for Memory Impairments and Neurological Disorders University of California, Irvine Irvine California USA; ^12^ Department of Neurology Washington University in St Louis St Louis Missouri USA; ^13^ Departments of Radiology and Neurology University of Washington Seattle Washington USA; ^14^ Department of Community and Behavioral Health, Elson S Floyd College of Medicine Washington State University Spokane Washington USA; ^15^ Department of Psychiatry University of Cambridge Cambridge Cambridgeshire UK; ^16^ Cambridgeshire & Peterborough NHS Foundation Trust Cambridge Cambridgeshire UK

**Keywords:** Alzheimer's disease, cognition, cognitive variability, dementia, dispersion, Down syndrome, intellectual disability, neuroimaging, resilience

## Abstract

**INTRODUCTION:**

Adults with Down syndrome (DS) are at risk for Alzheimer's disease (AD), yet identifying the preclinical phase remains challenging. Intraindividual cognitive variability (IICV) may be a sensitive marker of early AD‐related changes but remains understudied in DS.

**METHODS:**

Adults from the Alzheimer's Biomarker Consortium–DS (ABC‐DS) study (*N* = 460, mean age 43.3 years; 45.7% female) were included. Generalized linear models examined whether baseline IICV predicted incident mild cognitive impairment (MCI)/dementia, cognitive decline, and amyloid and tau positron emission tomography outcomes, adjusting for demographics, intellectual disability, apolipoprotein E ε4, site, assessment interval, and mean cognitive performance, with Bonferroni correction.

**RESULTS:**

Greater IICV predicted incident MCI/dementia (odds ratio = 4.63 to 5.13, *p* < 0.05), greater amyloid burden, early tau accumulation, and higher tau across Braak stages, independent of mean cognition. Exploratory analyses suggested sex‐specific interactions with tau outcomes.

**DISCUSSION:**

IICV is a sensitive marker of dementia risk and cognitive resilience in DS, with potential utility for secondary prevention and trial enrichment.

## Highlights

- IICV predicts dementia risk in adults with DS.
- Higher IICV is associated with amyloid burden and tau PET pathology.
- Cognitive variability detects clinical vulnerability before overt cognitive decline.
- Findings link cognitive heterogeneity to AD neuropathology in DS.
- Cognitive variability may aid risk stratification and trial enrichment in DS.

## 1 BACKGROUND

Down syndrome (DS) is genetically linked to Alzheimer's disease (AD),^1^ primarily due to the triplication of the amyloid precursor protein gene (APP) on chromosome 21, which, due to a gene‐dose effect, leads to dysregulation of the amyloid pathway.^2^ As a result, nearly all individuals with DS develop AD neuropathology,^3^ making this group an important population of interest for AD prevention and treatment trials and likely to benefit the most from such interventions. However, despite the stereotypical progression of the pathology, the onset of dementia and preclinical symptoms is variable. A recent meta‐analysis found that the mean age at dementia onset was 53.8 years,^4^ but there is a variation ranging from 30 to 70 years, highlighting the need for personalized diagnosis, treatment, and prognosis. From a clinical trials perspective, identifying sensitive and specific early markers of clinical decline is essential for participant selection and outcome measurement. The amyloid cascade hypothesis underscores the need for interventions during the preclinical phase, but challenges in accurately identifying the preclinical phase mean that existing trials face hurdles in selecting participants at high imminent risk for progression.

Studies on the utility of intraindividual cognitive variability (IICV) as a sensitive marker for AD preclinical changes are promising.^5^, ^6^, ^7^ IICV is the variability in a person's performance across cognitive measures and/or tasks administered at a single time point or in a short period of time. Unlike traditional cognitive approaches that focus on normative comparisons or longitudinal cognitive decline, IICV offers a person‐centered measure that may reflect subtle neuropathology or system instability.^8^ Increased IICV is linked to chronic brain pathology of various etiologies^9^ that may lead to higher dementia risk. Moreover, in studies in non‐DS populations, IICV has predictive value for incident mild cognitive impairment (MCI) and AD, comparable to well‐established biomarkers such as cerebrospinal fluid ptau181 and amyloid beta (A β) 40 and 42 peptide biomarkers,^6^ and has the potential to be a novel non‐invasive marker for prodromal dementia.^6^, ^9^, ^10^, ^11^, ^12^ Despite these promising findings, IICV has not been systematically investigated in adults with DS. If IICV can reliably predict time to dementia progression in individuals with DS, it could aid in stratifying risk, screening, and selecting individuals who would benefit most from new treatments and from clinical trials. It may also serve as a unique outcome measure for AD treatment trials with this population.

This study aims to characterize the relationship between IICV, AD biomarkers (including amyloid positron emission tomography [PET] and tau PET), and subsequent dementia onset in adults with DS. We hypothesized that higher baseline IICV would be associated with concurrent and subsequent AD‐biomarker positivity and predict future cognitive decline and dementia. Given the earlier clinical presentation of both executive dysfunction and memory impairment in DS‐related dementia,^13^, ^14^, ^15^ we anticipated that IICV capturing variability across these combined domains would show the strongest associations with disease progression.

## 2 METHODS

### 2.1 Study setting

#### 2.1.1 Study ethics

**RESEARCH IN CONTEXT**

1. **Systematic review**: We searched PubMed, PsycINFO, medRxiv, and ClinicalTrials.gov from inception to January 20, 2026, for studies on cognitive variability and AD in DS. Search terms included “intraindividual cognitive variability,” “cognitive variability,” and “cognitive dispersion” combined with “Down syndrome” and “Alzheimer” or “dementia.” No prior studies specifically examined IICV in individuals with DS.
2. **Interpretation**: This longitudinal study is the first to demonstrate that baseline IICV predicts amyloid burden, tau pathology, and incident dementia in adults with DS, identifying IICV as a potential indicator of disease progression and clinical risk.
3. **Future directions**: Future studies should evaluate its utility for prevention trial selection and determine its predictive performance relative to established biomarkers. In addition, further research is needed to elucidate the biological mechanisms linking cognitive variability, resilience, and AD pathology and to establish its generalizability across diverse DS populations.

This study involved the analysis of data collected from the Alzheimer's Biomarker Consortium‐DS (ABC‐DS) study, with Institutional Review Board‐approved protocols and written informed consent.

#### 2.1.2 ABC‐DS study

The ABC‐DS is a multi‐center cohort composed of participants with DS over 25 years of age recruited in the United States and the United Kingdom.^16^ The protocol involves a baseline visit and approximately 16‐month follow‐ups. It includes neuropsychological assessment, amyloid and tau PET, and consensus classification of dementia: (1) cognitively stable, (2) MCI‐DS, (3) dementia, and (4) not determined.

### 2.2 Study sample

We included data from the baseline and the third follow‐up visits (approximately 48 months apart), with a total of 460 participants at baseline who had complete cognitive data required for this analysis and 275 participants at follow up. For analyses of incident MCI/dementia and cognitive decline, individuals classified as having MCI (*n* = 57) or dementia (*n* = 37) or unable to determine (*n* = 11) at baseline were excluded. The procedure and instruments are briefly described below and have been fully described elsewhere.^16^, ^17^

### 2.3 Procedures

#### 2.3.1 Cognitive assessment

Neuropsychological assessments were completed within 3 months of the neuroimaging. The neuropsychological battery included the DS Mental Status Examination (DSMSE),^18^ a measure of general cognition; the Modified Cats & Dogs Task,^19^ a measure of executive function (response inhibition and working memory); the Cued Recall Task,^20^ a measure of cued retrieval of memory and processing speed; and the Rivermead Behavioral Memory Test,^21^ a measure of visual, verbal, recall, recognition, immediate, delayed, and prospective memory. Cognitive decline was defined as a decrease in DSMSE score from baseline to follow‐up exceeding one standard deviation from the mean change within each intellectual disability (ID) level (mild, moderate, severe), thereby accounting for group‐specific change in performance.

#### 2.3.2 Level of intellectual disability

Participants’ premorbid intellectual disability (mild, moderate, severe/profound) was determined using standardized intelligence quotient scores or documented medical records from best level of functioning.

#### 2.3.3 MCI/AD‐diagnosis status

A consensus diagnosis by experienced DS clinicians and study staff familiar with each participant classified participants as cognitively stable, MCI, dementia, or indeterminate by a consensus panel following the recommendations of the American Association on Mental Retardation‐International Association for the Scientific Study of Intellectual Disabilities (AAMR‐IASSID) Working Group for the Establishment of Criteria for the Diagnosis of Dementia in Individuals with Developmental Disabilities.^16^ The panel reviewed participants’ medical and psychiatric history as well as mean cognitive scores and had access to routine clinical lab results such as glucose, cholesterol, and thyroid‐stimulating hormone, but were blind to neuroimaging, genetic (apolipoprotein E, APOE), omics, and IICV data. The category of “unable to be determined” was assigned when the panel could not classify the participant into any diagnostic category based on the available data, which typically occurred due to insufficient information regarding changes in function or the presence of medical or psychiatric confounders at the time of the evaluation.

#### 2.3.4 PET scan

PET imaging was conducted at multiple participating centers with harmonized protocols. Scans were acquired on various PET scanner models, including ECAT HR+, four‐ring Biograph mCT, and Signa PET/MR, with injected doses of approximately 15 mCi for Pittsburgh Compound B (PiB) and 10mCI for florbetapir (FBP). PiB scans were typically acquired 50 to 70 min after injection, and FBP scans were acquired 80 to 100 min after injection (four 5‐min frames per scan). Using Statistical Parametric Mapping 12 (SPM12), PET frames were realigned to correct for motion and averaged to form a three‐dimensional image. Images were spatially normalized to MNI152 space via a DS‐specific PET template to account for anatomical differences in this population. T1‐weighted magnetic resonance imaging (MRI) scans were used to assist with spatial normalization and anatomical reference. MRI scans were acquired on 3T scanners using GE Signa 750, GE Signa PET/MR, and Siemens Trio or Prisma and processed using FreeSurfer (version 5.3.0) for region‐of‐interest definition. Standardized uptake value ratio images were generated using gray matter cerebellum as the reference.^22^ Amyloid burden was quantified using a Centiloid scale.^23^ Participants were scheduled for longitudinal imaging every 2 to 3 years; some participants completed multiple PiB of FBP scans, enabling assessment of longitudinal amyloid and tau burden and accumulation. In total, all available PiB and FBP scans from the full ABC‐DS cohort at the two time points considered for this study were included.

Amyloid burden was quantified on the Centiloid scale,^23^ and amyloid chronicity (“amyloid age”)^22^ was derived using the Sampled Iterative Local Approximation (SILA),^24^ which estimates a prototypical longitudinal amyloid trajectory; 18 Centiloids marks amyloid positivity onset (amyloid age = 0)^22^ (Figure S1). Disease‐stage sensitivity was assessed using IICV associations with amyloid age corresponding to the expected tau rise (2.5 years after amyloid onset).^25^

#### 2.3.5 Other measures

Sociodemographic data available for the ABC‐DS study were included in this analysis. Sex (male/female), race (White, Black/African American, American Indian/Alaskan Native, Asian, Native Hawaiian/Other Pacific Islander, or unknown), and ethnicity (Hispanic/Latino or non‐Hispanic/Latino) were self‐reported. APOE ε4 status was determined genetically. Time latency was defined as days between baseline and follow‐up. Trisomy 21 was confirmed through karyotyping (full, mosaic, translocation, and other variants).

### 2.4 Statistical approach

#### 2.4.1 Measures of IICV

Baseline within‐person cognitive variability was calculated by z‐transforming raw scores using the stable cognition group as reference,^6^ then computing individual standard deviation across z‐scores, as described in previous work.^5^, ^26^ Three IICV measures were created: (1) IICV‐Memory, using scores from the Cued Recall, Rivermead and memory score from the DSMSE; (2) IICV‐Executive, using the Modified Cats & Dogs Task, false positives from Rivermead and intrusions from Cued Recall; and (3) IICV‐Mem/Exec (memory and executive function), using all measures included in IICV‐Memory and IICV‐Executive. Each cognitive measure was z‐standardized prior to combination, and all measures contributed equally to the composite IICV. Table S1 shows the tests and subtests included in each IICV index, as well as the type of IICV they represent (within or across domain/test). Scores from the executive domain were multiplied by negative 1, so that higher scores consistently reflect better performance across all tests. In addition to within‐domain indices (memory IICV and executive IICV), we derived a cross‐domain IICV index combining memory and executive measures. This distinction reflects different conceptualizations of IICV, including variability within specific cognitive domains and variability across domains.^9^ Within‐domain indices capture heterogeneity in performance within memory or executive functioning, whereas the cross‐domain index captures dispersion between cognitive domains. This approach was chosen to examine whether early AD‐related cognitive changes in DS were better reflected by domain‐specific disruption or by broader cross‐domain divergence, given that early disease processes may differentially affect memory and executive systems.^13^, ^14^, ^15^

A higher IICV index represents greater variability across the cognitive measures included in each IICV index, and lower scores indicate more consistency across measures. IICV values were examined for outliers, and participants with values more than three standard deviations below the mean who demonstrated floor‐level performance on one or more of the tests were excluded from the respective analysis (Mem/Exec, *n* = 4; Mem, *n* = 3, Exec, *n* = 4), as these values may reflect premorbid intellectual disability rather than meaningful variability.

#### 2.4.2 Statistical analysis

Descriptive analyses of the demographic and clinic variables are reported as absolute and relative frequencies or as means and standard deviations. Generalized linear models assessed associations between IICV and outcomes, adjusting for age, sex, intellectual disability, APOE ε4 status, site, and, where applicable, follow‐up interval. Analyses predicting incident MCI/dementia were conducted in participants who were cognitively stable at baseline. Model assumptions were evaluated, and scatterplots were used to confirm homoscedasticity and linearity. Statistical significance was set as *α* = 0.05, with Bonferroni correction for multiple comparisons. Significant associations were further adjusted for mean cognitive z‐score to assess IICV's independent contribution, using standardized values to facilitate effect‐size comparisons. Exploratory sex‐by‐IICV interactions were tested by including interaction terms between sex and each IICV measure in the respective regression models alongside all relevant covariates. Analyses were conducted using IBM SPSS Statistics version 29 (Windows) and R version 4.5 (R Foundation for Statistical Computing, Vienna, Austria).

## 3 RESULTS

### 3.1 Participant characteristics

Mean baseline age was 43.27 years (range 25 to 81), 45.7% were female. Most participants were White (96.3%), and non‐Hispanic or Latino (94.8%), 49.7% (*n* = 229) had mild premorbid intellectual disability, and 23.3% (*n* = 107) were APOE ε4 carriers. At baseline, 72.2% were cognitively stable, 12.4% had MCI‐DS, 8% had dementia, and 2.4% were indeterminate. Follow‐up data were available for 59.7% (mean 40 ± 8 months, range 20 to 60, Table 1 and Table S2).

**TABLE 1:** Demographic characteristics of the participants at baseline and follow‐up of approximately three years apart.

	Baseline (*N* = 460)	Follow‐up (*N* = 275)
Age, mean (SD) / range	43.27 (9.97)/ 25−81	47.27 (9.53)/ 28−75
Self‐reported sex, *n* female (%)	210 (45.7)	124 (45.1)
Race, *n* (%) Black/ African American	6 (1.3)	3 (1.1)
American Indian/ Alaskan Native	1 (0.2)	0 (0.0)
Asian	6 (1.3)	3 (1.1)
White	443 (96.3)	266 (96.7)
Native Hawaiian/ Other Pacific Islander	0 (0.0)	0 (0.0)
Unknown	4 (0.9)	3 (1.1)
Ethnicity, *n* Hispanic or Latino (%)	24 (5.2)	11 (4.0)
Karyotype ^1^ Trisomy 21	409 (88.9)	247 (89.8)
Mosaicism	17 (3.7)	12 (4.4)
Translocation	21 (4.6)	12 (4.4)
Other	1 (0.2)	1 (0.4)
Degree of intellectual disability, *n* (%)^2^ Mild	229 (49.9)	150 (54.5)
Moderate	191 (41.5)	100 (36.4)
Severe	39 (8.5)	25 (9.1)
*APOE e4* allele carrier, *n* (%)^3^	107 (23.3)	62 (22.6)
Consensus diagnosis, n (%) No Dementia/ MCI‐DS	355 (72.2)	184 (66.9)
MCI‐DS	57 (12.4)	27 (9.8)
Dementia	37 (8.0)	56 (20.4)
Unable to be determined	11 (2.4)	8 (2.9)
Time latency in days (follow‐up minus baseline), mean (SD/ range)	—	1194.95 (235.30)/ 622‐1835
**Cognitive variability measures**		
Intraindividual cognitive variability^#^, mean (SD)/ range		
*Memory* (*n* = 346)	0.83 (0.72) / 0.04–3.21	
*Executive function* (*n* = 320)	0.83 (0.71) / 0.03‐5.11	
*Memory/ executive* (*n* = 320)	0.87 (0.61) / 0.08–3.09	
**Neuroimaging findings**		
Amyloid‐β measured in centiloids, mean (SD)/ range (Baseline n = 239, Follow‐up *n* = 87)	30.91 (38.39)/ −19.19 −180.11	19.14 (26.77)/ −7.60‐109.21
Amyloid age, years	−2.42 (10.04)/ ‐37.50 –13.72	−4.16 (10.21)/ −33.50 –10.29
Amyloid age ≥ 2.5, n (%)	78 (32.0)	20 (23.0)
**PET‐tau Neurofibrillary Braak tangle stage**		
(Baseline *n* = 141, Follow‐up *n* = 80) I‐II	1.18 (0.19)	1.20 (0.18)
III‐IV	1.14 (0.18)	1.15 (0.20)
V‐VI	1.08 (0.21)	1.09 (0.19)

*Note*: ^1^Twelve with missing data at baseline and three at follow‐up; ^2^ Two missing at baseline; ^3^Twenty missing at baseline and one at follow‐up; ^#^IICV denotes within‐person test variability measured as the standard deviation of tests/subtest z‐scores. Z‐scores were standardized using mean and standard deviation values derived from participants with stable cognition only. Increased variability theoretically denotes worse cognitive function.

### 3.2 Baseline IICV and risk of cognitive decline and dementia at follow‐up

Table 2 summarizes associations between IICV and incident MCI/dementia and cognitive decline. After excluding individuals with MCI/dementia or unable to determine, greater IICV‐Mem/Exec was strongly associated with incident MCI/dementia (odds ratio [OR] = 25.20, 95% confidence interval [CI] [4.06, 156.37]), followed by IICV‐Exec (OR = 8.71, 95% CI [2.29, 33.04]). IICV‐Mem was not significant. IICV‐Exec and IICV‐Mem/Exec explained 14.1% and 18.7% of variance and classified ∼89.2% of cases, whereas age alone explained 38%. No IICV indices were associated with cognitive decline after Bonferroni correction.

**TABLE 2:** Separate logistic regression models assessing the associations between baseline measures of intra individual cognitive variability (IICV^#^) and two follow‐up outcomes: (1) mild cognitive impairment (MCI) or dementia diagnosis, and (2) a decline of at least one standard deviation below the mean change in total DSMSE score from baseline, stratified by level of intellectual disability (ID). Participants at baseline with MCI, dementia or unable to determine were excluded from the analyses. Statistics reported in the table refer to the cognitive variable after adjusting for age, sex, level of intellectual disability, presence of APOE‐*e*4, site and time latency between baseline and follow‐up assessment.

		95% Confidence Interval			*Bonferroni Adjusted p*
	Odds ratio	Lower bound	Upper bound	*p*‐value	
**Dependent variable: MCI or dementia diagnosis at follow‐up**					
***Predictor***: ***IICV‐Mem*** *(n* =* 202, 16 MCI and 8 dementia)*	1.774	0.607	5.184	0.295	1.000
***IICV‐Exec*** *(n* = *194, 16 MCI, 6 dementia)*	8.712	2.297	33.047	0.001	**0.036**
***IICV‐Mem/ Exec*** *(n* = *194, 16MCI, 6 dementia)*	25.202	4.062	156.375	<0.001	**<0.036**
Dependent variable: 1SD below the mean change in DSMSE* *(from baseline to follow‐up stratified by level of ID)* ***Predictor***:					
***IICV‐ Mem***	2.487	0.860	7.191	0.092	1.000
***IICV‐ Exec***	6.204	1.399	27.517	0.016	0.576
***IICV‐ Mem/ Exec***	6.160	1.075	35.308	0.041	1.000

*Note*: Key: ^#^IICV denotes within‐person test variability measured as the standard deviation of tests/subtest z‐scores. Increased variability theoretically denotes worse cognitive function; *cut‐off of 1SD above the mean change according to level of intellectual disability (ID): for mild ID decline ≥ 9 points, for moderate ID decline ≥ 6 points, and for severe ID decline ≥ 111points; ^τ^ mild ID *n* = 11, moderate ID *n* = 9, severe ID *n* = 1.

### 3.3 Associations between baseline IICV and Aβ‐PET

Table 3 shows that greater IICV was cross‐sectionally associated with higher estimated amyloid chronicity, with all three indices remaining significant after Bonferroni correction and the strongest effect observed for the combined IICV Mem/Exec (*β* = 0.375). At follow‐up, IICV‐Mem/Exec and IICV‐Exec were nominally associated with amyloid chronicity, but these associations did not survive correction.

**TABLE 3:** Separate regression models evaluating the associations between baseline measures of intra individual cognitive variability (IICV) and amyloid‐β chronicity measured at two time points (baseline and follow‐up) adjusted for sex, level of intellectual disability, presence of APOEe4 and site. For measures at follow‐up, the model was also adjusted for time latency between baseline and follow‐up. Statistics reported in the table refer to the contribution of the cognitive predictors after controlling for the other variables in each model.

	Unstandardized coefficient	Standardized coefficient	95% Confidence interval for *B*			*Bonferroni adjusted p*
	*B*	*β*	Lower bound	Upper bound	*p*‐value	
**Dependent variable**:						
Baseline estimated amyloid chronicity (amyloid age), *n* = 228						
*IICV‐ Mem*	5.628	0.326	3.374	7.882	<0.001	**<0.036**
*IICV‐ Exec*	5.589	0.270	2.909	8.270	<0.001	**<0.036**
*IICV‐ Mem/ Exec*	7.855	0.375	5.117	10.593	<0.001	**<0.036**
Follow‐up estimated amyloid chronicity (amyloid age), *n* = 85						
*IICV‐ Mem*	5.638	0.210	−0.510	11.785	0.072	1.000
*IICV‐ Exec*	5.870	0.252	0.306	11.433	0.039	1.000
*IICV‐ Mem/ Exec*	8.785	0.327	2.309	15.260	0.008	0.288

		95% Confidence Interval			*Bonferroni Adjusted p*
	Odds ratio	Lower bound	Upper bound	*p*‐value	
**Dependent variable**:					
Baseline amyloid age ≥ 2.5* (*n* = 228, 71 participants amyloid age ≥ 2.5)					
*Predictor*:					
*IICV‐Mem*	8.412	3.872	12.275	<0.001	**<0.036**
*IICV‐Exec*	5.457	2.544	11.704	<0.001	**<0.036**
*IICV‐Mem/ Exec*	13.361	5.169	34.538	<0.001	**<0.036**
Follow‐up amyloid age ≥ 2.5 (*n* = 85, 19 participants amyloid age ≥ 2.5)					
*Predictor*:					
*IICV‐ Mem*	3.805	0.826	17.521	0.086	1.000
*IICV‐ Exec*	4.359	1.103	17.217	0.036	1.000
*IICV‐ Mem/ Exec*	14.291	2.199	92.876	0.005	0.180

*Note*: Key: ^#^IICV denotes within‐person test variability measured as the standard deviation of tests/subtest z‐scores. Increased variability theoretically denotes worse cognitive function. *Amyloid age for estimated early tau increases (2.5 years following A+ onset, Zammit et al 2023).

IICV was also cross‐sectionally associated with increased odds of reaching an amyloid threshold linked to early tau accumulation (≥ 2.5 years)^25^ for all indices after Bonferroni adjustment, whereas longitudinal associations did not remain significant after correction.

### 3.4 Associations between baseline IICV and Tau pathology

Table 4 shows that, although all IICV indices were associated with tau PET measures before Bonferroni, only IICV‐Exec and IICV‐Mem/Exec remained significantly associated with baseline tau PET signal at Braak stages III–VI after correction (B = 0.111 to 0.196, *p *< 0.036). IICV‐Mem was nominally associated with tau at stages III–VI before correction (B = 0.115 to 0.113, *p *= 0.044 to 0.043). No baseline IICV associations with follow‐up Braak stages survived correction.

**TABLE 4:** Separate linear regression models assessing the associations between baseline measures of intra individual cognitive variability (IICV) and PET amyloid‐tau in Braak neurofibrillary tangle at two time points (baseline and follow‐up). All models were adjusted for age, sex, level of intellectual disability, presence of APOEe4 and site. For analyses considering follow‐up outcomes, the model was also adjusted for time latency between baseline and follow‐up. Statistics reported in the table refer to the contribution of the cognitive predictors after controlling for the other variables in each model.

	Unstandardized coefficient	Standardized coefficient	95% Confidence interval for *B*			*Bonferroni adjusted p*
	*B*	*β*	Lower bound	Upper bound	*p*‐value	
**Dependent variable**:						
Baseline PET‐tau Neurofibrillary Braak tangle stage, *n* = 136						
**I‐II**						
*IICV‐ Mem (n *= *136)*	0.072	0.186	0.007	0.137	0.031	1.000
*IICV‐ Exec (n* = *133)*	0.080	0.192	0.012	0.149	0.022	0.792
IICV‐ Mem/ Exec (*n* = 133)	0.113	0.248	0.033	0.193	0.006	0.216
III‐IV						
*IICV‐ Mem (n = 136)*	0.081	0.217	0.016	0.145	0.015	0.540
*IICV‐ Exec (n = 133)*	0.111	0.284	0.046	0.175	<0.001	**<0.036**
IICV‐ Mem/ Exec (*n* = 133)	0.151	0.355	0.076	0.226	<0.001	**<0.036**
V‐VI						
*IICV‐ Mem*	0.105	0.236	0.024	0.186	0.011	0.396
*IICV‐ Exec (n = 133)*	0.148	0.327	0.070	0.226	<0.001	**<0.036**
IICV‐ Mem/ Exec (*n* = 133)	0.196	0.396	0.105	0.287	<0.001	**<0.036**
Follow‐up PET‐tau Neurofibrillary Braak tangle stage, *n* = 78						
I‐II						
*IICV‐ Mem(n = 79)*	0.023	0.055	−0.073	0.118	0.638	1.000
*IICV‐ Exec (n = 79)*	0.033	0.079	−0.062	0.128	0.489	1.000
*IICV‐ Mem/ Exec (n = 79)*	0.046	0.097	−0.076	0.167	0.456	1.000
III‐IV						
*IICV‐ Mem (n = 79)*	0.115	0.241	0.003	0.226	0.044	1.000
*IICV‐ Exec (n = 79)*	0.020	0.042	−0.095	0.135	0.725	1.000
*IICV‐ Mem/ Exec (n = 79)*	0.125	0.229	−0.019	0.268	0.087	1.000
V‐VI						
*IICV‐ Mem (n = 79)*	0.113	0.253	0.004	0.223	0.043	1.000
*IICV‐ Exec (n = 79)*	0.026	0.057	−0.087	0.139	0.646	1.000
*IICV‐ Mem/ Exec (n = 79)*	0.138	0.270	−0.002	0.279	0.053	1.000

*Note*: Key: ^#^IICV denotes within‐person test variability measured as the standard deviation of tests/subtest z‐scores. Increased variability theoretically denotes worse cognitive function.

### 3.5 Sensitivity analyses

Sensitivity analyses using IICV computed from full‐sample z‐scores yielded consistent results.

### 3.6 Predictive value of IICV beyond mean cognitive scores

**FIGURE 1 alz71537-fig-0001:**
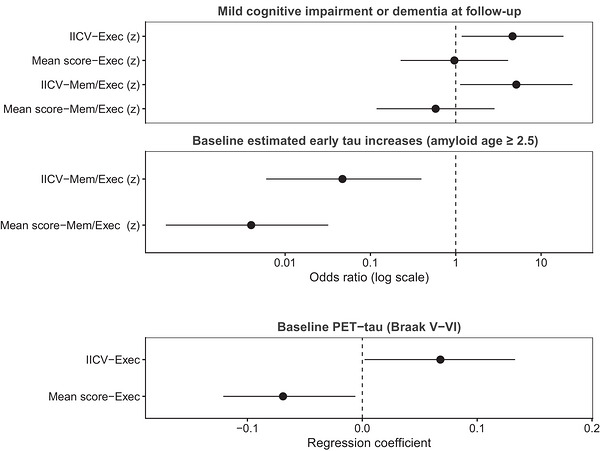
Incremental predictive value of intraindividual cognitive variability (IICV) beyond mean cognitive performance: forrest plot of standardized effect sizes. All models were adjusted for age, sex, level of intellectual disability, presence of APOEe4 and site. For analyses considering follow‐up outcomes, the model was also adjusted for time latency between baseline and follow‐up. Statistics reported in the figure refer to the contribution of the cognitive predictors when entered together in models after controlling for the other variables.

Regression models including IICV and the corresponding mean cognitive scores (z‐standardized) were adjusted for age, sex, level of intellectual disability, APOE ε4 status, site, and, for follow‐up outcomes, time latency (Figure 1). Higher IICV‐Exec (OR = 4.632, 95% CI [1.173, 18.298], *p *= 0.029) and IICV‐Mem/Exec (OR = 5.127, 95% CI [1.125, 23.363], *p *= 0.035) predicted increased odds of incident MCI/dementia, whereas mean scores were not significant. When modeled with mean performance, IICV‐Mem/Exec and mean Mem/Exec were associated with reduced risk of early tau accumulation (OR = 0.047, *p *= 0.005; OR = 0.004, *p *< 0.001).

IICV‐Exec was positively associated with stage V–VI (B = 0.068, *β* = 208, *p *= 0.043), and mean performance was also associated with tau across stages (*β* range = −0.295 to −0.904, all *p *< 0.05). Together, these findings indicate that IICV, particularly in executive domains, adds predictive value for tau accumulation beyond mean cognition (Tables S3–S4).

### 3.7 Exploratory interactions of sex versus IICV

Exploratory sex‐by‐IICV interactions were significant in nine of 36 models, all involving tau PET outcomes (Table S5). Significant interactions included IICV‐Mem across baseline and follow‐up Braak stages I–VI, IICV‐Exec for follow‐up stages III–VI, and IICV‐Mem/Exec for baseline stages III–IV (interaction coefficients −1.032 to 0.821, *p* = 0.002 to 0.029. Memory‐based IICV interactions indicated stronger associations in males, whereas executive‐based interactions were stronger in females. No significant interactions were observed for MCI/dementia, cognitive decline, or amyloid chronicity.

## 4 DISCUSSION

In 460 aging adults with DS, baseline IICV was significantly associated with follow‐up clinical status, including MCI/dementia risk, and with amyloid burden and tau PET measures. Consistent with prior work in the general population,^5^, ^27^, ^28^ these findings support IICV as a marker of dementia and neurodegeneration for individuals with DS, with potential use for risk stratification, trial selection, and intervention outcomes.

Baseline IICV was associated with greater estimated amyloid chronicity independent of demographics, supporting its potential as a sensitive marker in individuals with DS. Higher IICV across all indices was also strongly associated with surpassing an amyloid chronicity threshold (≥2.5 years) previously linked to early tau accumulation,^25^ suggesting IICV may mark the point at which amyloid drives downstream tau changes. This is particularly relevant in DS, where amyloid accumulation is nearly universal by mid‐adulthood,^3^ yet not all individuals develop dementia.^29^, ^30^ IICV may help identify individuals at higher risk for symptomatic progression. Those findings highlight the need for complementary biomarkers, reflecting synaptic dysfunction, cell death, or bioenergetic compromise. Larger studies are needed to examine temporal dynamics and IICV's potential as an early intervention outcome.

Individuals with greater IICV at baseline, particularly in executive functioning or memory‐executive domains, showed higher tau pathology in advanced Braak stages (III–VI), consistent with clinical dementia stages in DS.^31^ While some associations at follow‐up were suggestive, they did not survive multiple‐comparisons correction, possibly due to the limited sample size or absent longitudinal effects. These preliminary findings highlight potential links between early cognitive variability and tau accumulation, warranting further investigation in larger longitudinal studies clarifying temporal dynamics in this population.

IICV was not significantly associated with longitudinal DSMSE changes, suggesting it may index early clinical vulnerability even when measurable decline is undetectable within a 3‐year period, possibly due to DSMSE's limited sensitivity to subtle change. Conventional cognitive assessments capture quasi‐equilibrium performance rather than dynamic processes reflecting resilience or adaptive capacity. Decline may emerge later or be difficult to detect in individuals with pre‐existing intellectual disability. Consistent with prior work, behavior changes may precede measurable cognitive decline in DS.^14^, ^32^ Notably, IICV was associated with dementia diagnosis, amyloid burden, and tau pathology, supporting its potential as an early marker of emerging clinical impairment prior to overt cognitive decline. Associations with biological markers mitigate potential circularity in clinical diagnosis, where variability may be incorporated into adjudication. This pattern of associations, linking IICV to biomarkers and diagnosis but not measurable cognitive decline, suggests dissociation between underlying pathology and observable symptoms in early DS‐related AD, highlighting the roles of cognitive resilience.^33^ In previous work with DS, biological‐clinical discordance displayed a pattern consistent with resilience, with advanced pathology elative to clinical stage, in contrast to sporadic AD.^34^ Given early, near‐universal amyloid accumulation but variable dementia onset in DS, this population may be informative for studying compensatory mechanisms^35^ and clinical trial opportunities. Conceptually, increased IICV may reflect rising synaptic or network‐level noise,^8^ indexing inefficiencies in upstream cognitive processes rather than random error, analogous to synaptic mechanisms such as increased neurotransmitter release probability during long‐term potentiation.^36^ Deviations from optimal network states may signal compensatory responses to pathology. If error‐driven updating within these networks is impaired, then cognitive systems may expend disproportionate energy to maintain performance.^37^ This would result in increased variability without measurable decline. As neurodegeneration progresses and compensation fails, variability may decrease, reflecting reduced cognitive flexibility and reserve. This dynamic may explain the sensitivity of IICV as an early vulnerability marker in the absence of detectable decline.

Findings[Fig alz71537-fig-0001] indicate that IICV is a more sensitive predictor of future clinical outcomes than mean cognitive performance, particularly for identifying individuals at greater risk of MCI/dementia. When modeled jointly, only IICV, especially across combined memory and executive domains, remained significantly associated with future diagnosis, suggesting that IICV captures early risk stratification and intervention in clinical trials, even when mean cognitive scores remain within normal limits. Executive function IICV showed robust associations with tau pathology across Braak V–VI stages, independent of mean performance. However, associations between IICV and AD biomarkers varied by domain and disease stage, underscoring the need to disentangle domain‐specific effects. In some models, the direction of the associations reversed when mean scores were included. This may suggest that, when considered alongside mean scores, IICV provides an adjustment of the association attributable to the mean score, potentially attenuating it. Thus, the observed association between IICV‐Mem/Exec, mean performance, and reduced risk of early tau accumulation should be interpreted within the context of shared variance between variability and mean cognitive performance. Future longitudinal studies should examine how IICV evolves over time and whether domain‐specific variability differentially predicts progression from preclinical to symptomatic AD. IICV may follow a non‐linear trajectory, increasing in early stages of neurodegeneration and declining as dementia progresses, consistent with a loss of cognitive flexibility and reserve. Future studies should test whether IICV reflects synaptic or network‐level noise using multimodal neuroimaging and electrophysiological measures to better characterize its neural basis.

The domain‐specific associations observed between IICV and AD biomarkers may reflect differential vulnerability of underlying neural systems. Variability in executive and memory/executive domains showed the most robust associations with amyloid and tau, potentially reflecting early vulnerability of frontal and large‐scale association system.^38^, ^39^ In contrast, memory‐related variability may be more closely linked to medial temporal lobe pathology.^40^ These complementary indices allowed assessment of within‐domain and cross‐domain variability, reflecting early heterogeneous cognitive change in DS, where executive and memory systems may be differentially affected. IICV may reflect domain‐specific and network‐level manifestations of early AD‐related neurobiological changes.

Given that Aβ accumulation progresses more rapidly in individuals with DS, as observed also in autosomal dominant AD, compared to late‐onset AD,^41^, ^42^, ^43^ likely due to differences in age onset, identifying predictive markers of disease progression after amyloid positivity is crucial. Such markers can help clarify variability in clinical trial outcomes by distinguishing between treatment effects and individual differences in the natural trajectory of amyloid buildup or resilience to downstream neurodegeneration. Ultimately, integrating IICV into the study of AD‐DS may enhance our understanding of disease heterogeneity, enable more precise early detection, and inform targeted strategies for dementia prevention and intervention.

Although exploratory, interactions between sex and IICV in particular for PET tau outcomes may indicate that sex moderates the relationship between cognitive variability and tau pathology in adults with DS. Similarly, a recent study in type 1 diabetes observed sex‐specific interactions with plasma biomarkers.^44^ These findings suggest that sex may differentially influence the associations of cognitive variability and markers of neurodegeneration in different populations. Prior studies with participants with DS suggested sex differences in AD biomarkers and cognition. *Post mortem* analyses showed elevated tau pathology in females with DS.^45^ Consistently, a study of age, sex, and neuropathology indicated that women with DS may show more advanced tau stages.^46^ While longitudinal cohort studies show no overall sex differences in AD risk, they indicate longer dementia duration in females^47^ and domain‐specific cognitive differences, including lower episodic memory, in males with DS.^48^ These findings provide a biological context consistent with our exploratory analyses, but replication in different cohorts is needed to confirm whether sex systematically modifies IICV–biomarker associations. Potential mechanisms include sex differences in hormones, neuroinflammation, and vascular risk, which may affect amyloid and tau trajectories. Clinically, these findings support considering sex as a stratification factor in future studies and trials, as it may influence biomarker–cognition relationships.

The strengths of this study include the use of the ABC‐DS cohort, providing a large, well‐characterized, multicenter sample of individuals with DS, focus on an understudied high‐risk population, and a rigorous statistical approach accounting for data structure and multiple comparisons. The study's limitations include limited sample sizes for some analyses, particularly incident dementia and longitudinal PET models, and restriction to two time points over ∼40 months, despite evidence that IICV may be informative up to a decade before clinical AD onset, underscoring the need for longer follow‐up. A small number of participants with floor‐level performance were excluded from IICV analyses, limiting generalizability to those with severe impairment. Finally, the lack of racial and ethnic diversity (∼95% White, non‐Hispanic) further limits generalizability and may overlook differences in social, environmental, and biological factors. Future studies should improve diversity through targeted and community‐engaged recruitment and multi‐site collaborations.

IICV represents a low‐burden, easily scalable cognitive marker, particularly valuable in low‐resourced settings with limited access to advanced biomarkers. Inclusion of individuals with DS in biomarker research supports the detection of neurodegenerative changes at asymptomatic stages, while early cognitive markers such as IICV may inform mechanisms of resilience and guide interventions aimed at optimizing cognitive function and reducing long‐term disability. At present, however, there are no established clinical cut‐offs or standardized assessment frequencies for IICV, and further validation is needed before its implementation in routine clinical practice.

This study demonstrates that IICV is a sensitive AD‐risk marker in individuals with DS, with higher baseline IICV associated with incident MCI/dementia, greater amyloid chronicity, and elevated tau. Many associations remained after adjusting for mean cognition, highlighting the unique contribution of IICV beyond traditional metrics. Findings support its use in DS and dementia research, early identification, trial selection, and studies of mechanisms linking cognitive variability, resilience, and AD pathology.

## COLLABORATORS

^*^ ABC‐DS Investigators:

Beau M. Ances, MD, PhD; Howard F. Andrews, PhD; Karen Bell, MD; Rasmus M. Birn, PhD; Adam M. Brickman, PhD; Peter Bulova, MD; Jeff Burns, MD; Amrita Cheema, PhD; Kewei Chen, PhD; Bradley T. Christian, PhD; Isabel Clare, PhD; Ann D. Cohen, PhD; Eric W. Doran, MS; Tatiana M. Foroud, PhD; Benjamin L. Handen, PhD; Jordan Harp, PhD; Sigan L. Hartley, PhD; Elizabeth Head, PhD; Denise Head, PhD; Christy Hom, PhD; Lawrence Honig, MD; Milos D. Ikonomovic, MD; Sterling C Johnson, PhD; M. Ilyas Kamboh, PhD; David Keator, PhD; Julia K. Kofler, MD; William Charles Kreisl, MD; Sharon J. Krinsky‐McHale, PhD; Florence Lai, MD; Patrick Lao, PhD; Charles Laymon, PhD; Joseph Hyungwoo Lee, PhD; Ira T. Lott, MD; Victoria Lupson, PhD; Mark Mapstone, PhD; Davneet Singh Minhas, PhD; Neelesh Nadkarni, MD; Sid O'Bryant, PhD; Deborah Pang, MPH; Melissa Petersen, PhD; Julie C. Price, PhD; Lauren Ptomey, PhD; Margaret Pulsifer, PhD; Michael S. Rafii, MD PhD; Herminia Diana Rosas, MD; Frederick Schmitt, PhD; Nicole Schupf, PhD; Wayne P. Silverman, PhD; Dana L. Tudorascu, PhD; Rameshwari Tumuluru, MD; Badri Varadarajan, PhD; Michael A. Yassa, PhD; Shahid Zaman, MD PhD; Fan Zhang, PhD

## CONFLICT OF INTEREST STATEMENT

EH receives book royalties from Elsevier Press and consulting fees from Cyclo Therapeutics, Alzheon, and Elsevier and serves on advisory boards for Duke University, UC Davis, Washington University in St. Louis, and Kansas University Medical Center. JC receives consulting fees from Expert Connect. SZ receives royalties from Pavillon Publishing. MM has royalties from the University of Rochester, consulting fees from NovoGlia and Ireneo Health, serves on advisory boards for Brain Neurotherapy, the Davis Phinney Foundation for Parkinson's, Alzheon, the Super Aging Research Initiative, the ACT Trial (Efficacy and Mechanisms of Combined Aerobic Exercise and Cognitive Training in Mild Cognitive Impairment), and Ireneo Health. BH receives book royalties. BC receives consulting fees from Alnylam, Lathes, and AVID Radiopharmaceuticals. All other co‐authors declare no conflicts of interest. Author disclosures are available in the Supporting Information.

## CONSENT STATEMENT

All human subjects provided informed consent.

## Supporting information



Supporting Information

Supporting Information

## Data Availability

The data that support the findings of this study are openly available in the ABC‐DS database (https://ida.loni.usc.edu/login.jsp?project = ABCDS)
