# Supplementary material for: Intraindividual cognitive variability predicts amyloid beta, tau PET, and dementia conversion in Down syndrome: a potential marker of cognitive resilience
Source: Alzheimers Dement. 2026 Jun 6;22(6):e71537. doi: 10.1002/alz.71537 (PMC13242613; doi:10.1002/alz.71537)
Supplement: Supplementary file 1 — Supporting Information [file ALZ-22-e71537-s001.docx]

| Supplemental table 1. Tests and subtests included in the intraindividual cognitive variability (IICV) measures | | | |
| --- | --- | --- | --- |
| **Cognitive domain** | **Test** | **Measures included** | **Type of IICV** |
| Memory | Cued Recall  Rivermead Picture Recognition Delay  Down syndrome Mental Status Examination (DSMSE) | - Total learning free and cued recall - Total correct - Memory score (location) | Across-test  Within-domain |
| Executive function* | Cats & Dogs  Rivermead Picture Recognition Delay  Cued Recall | - Naming time (multiplied to minus 1) - Switching time (multiplied to minus 1) - Total false positive (multiplied to minus 1) - Total intrusions (multiplied to minus 1) | Across-test  Within-domain |
| Memory/ Executive | All tests included in IICV Memory and IICV Executive Function | All measures included in IICV Memory and IICV Executive Function | Across-test  Across domain |

**Supplemental Figure 1.** Sampled iterative local approximation (SILA) modeled fit of estimated amyloid chronicity. Each line represents one participant; points represent longitudinal data.


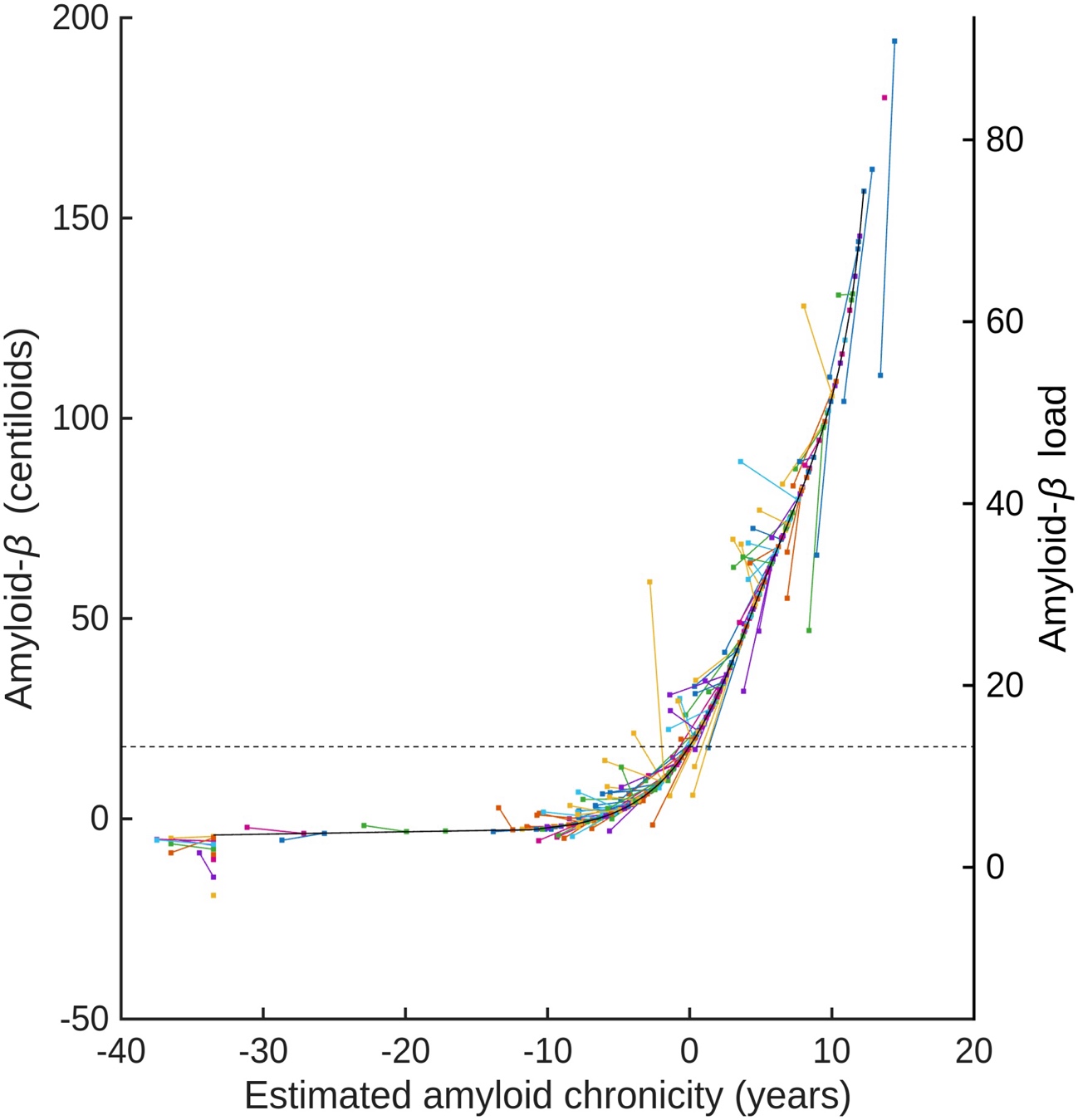


Amyloid chronicity (“amyloid age”)^14^ using the Sampled Iterative Local Approximation (SILA) algorithm. This method estimates a prototypical longitudinal trajectory of amyloid accumulation and aligns individuals along this trajectory based on their amyloid levels. In doing so, it redefines their chronological age as an “amyloid age,” reflecting where along the estimated trajectory of amyloid accumulation each subject lies. Negative values of amyloid age denote the expected time in years before onset of amyloid positivity, and positive values denote the expected time in years since onset. Hence, amyloid age serves as a measure of disease stage, especially during the earlier stages of the disease.

| **Supplemental table 2. Cognitive findings.** | |  |
| --- | --- | --- |
|  | Baseline (N = 460) | Follow-up (N = 275) |
| **Cognitive test scores**, mean (SD)/ range |  |  |
| **Memory** |  |  |
| *Cued Recall* - learning free and cued, score range 0-36, (n=444) | 28.32 (10.02) / 0 – 36 |  |
| *Rivermead Picture Recognition Delay, score range 0-10* (n=355) | 8.50 (2.38) / 0 – 10 |  |
| *DSMSE*-memory score^λ^ (n=460), | 4.52 (1.74) / 0 – 6 |  |
| **Executive function and processing speed** |  |  |
| *Cats & Dogs* |  |  |
| Naming time (n=444) | 17.38 (11.45) / 6 – 90 |  |
| Switching time (n=425) | 26.55 (15.01) / 7.2 – 90 |  |
| *Cued Recall* Intrusions (n=443) | 6.50 (7.63) / 0 – 35 |  |
| *Rivermead Picture Recognition Delay*- False positive (n= 354) | 3.62 (3.95) / 0 – 10 |  |
| **General Cognition** |  |  |
| *DSMSE*- Total Score, range 0-85 | 51.00 (14.32) / 4 – 84  (n = 460) | 58.07 (17.83) / 0 – 85  (n = 259) |
| **Other cognitive measures,** mean (SD)/ range or n (%) | |  |
| **Change in DSMSE- Total Score**^λ^ (follow-up minus baseline) | |  |
| Total sample with data (n=259)  *By level of intellectual disability (ID)* |  | 4.15 (11.46)/ -34 - 50 |
| Mild ID (n = 143) |  | 4.53 (12.44) / -34– 48 |
| Moderate ID (n = 93) |  | 4.45 (10.18)/ -15– 50 |
| Severe ID (n = 23) |  | 0.52 (9.65)/ -22– 20 |
| **One SD above mean change (decline) according to level of intellectual disability*** |  | 33 (12.74%)^τ^ |
| Key: SD, standard deviation; *cut-off of 1SD above the mean change according to level of intellectual disability (ID): for mild ID decline ≥ 17 points, for moderate ID decline ≥ 15 points, and for severe ID decline ≥ 11 points; ^λ^DSMSE memory and total scores were calculated using the method that excludes the task memory for object, in order to maximize the number of participants; ^τ^ mild ID n = 19, moderate ID n = 11, severe ID n= 3. | | |

| **Supplemental table 3. Logistic regression models in which IICV remained a significant predictor after inclusion of mean baseline scores.** IICV and mean scores were z standardized. All models were adjusted for age, sex, intellectual disability, presence of APOEe4, site and mean score for the respective cognitive tests included in the IICV measure. Models predicting outcomes at follow-up included time latency between baseline and follow-up. Regression involving amyloid age did not include age, as this metric already accounts for age. Statistics reported in the table refer to the contribution of the cognitive variable predictors after controlling for the other variables in each model. | | | | |
| --- | --- | --- | --- | --- |
|  | **Odds ratio** | **95% Confidence**  **Interval** | | ***p*** |
|  |  | **Lower bound** | **Upper bound** |  |
| **Dependent variable:**  **MCI or Dementia diagnosis at follow-up**  (N=181, 10 participants with MCI or dementia) | | | | |
| ***Predictor:****  ***IICV-Exec*** *(z-standardized)* | 4.632 | 1.173 | 18.298 | **0.029** |
| *Mean score for executive domain (z-standardized)* | 0.964 | 0.226 | 4.109 | 0.960 |
|  |  |  |  |  |
| ***IICV-Mem/ Exec*** *(z-standardized)* | 5.127 | 1.125 | 23.363 | **0.035** |
| *Mean score for memory/ executive domains (z-standardized)* | 0.581 | 0.118 | 2.849 | 0.503 |
| ***Dependent variable:***  ***Estimated early tau increases at baseline (amyloid age* ≥ 2.5)**  (N=228, 71 participants amyloid age≥ 2.5) |  |  |  |  |
| ***Predictor*:***  ***IICV- Mem/Exec*** *(z-standardized)* | 0.047 | 0.006 | 0.396 | **0.005** |
| *Mean score for memory/ executive domains (z-standardized)* | 0.004 | 0.0004 | 0.032 | **<0.001** |
| Key: ^#^IICV denotes within-person test variability measured as the standard deviation of tests/subtest z-scores. *Statistics reported in the table refer to IICV variable after adjusting for the other variables in the model. MCI, mild cognitive impairment | | | | |

| **Supplemental table 4. Linear regression models in which IICV remained a significant predictor after inclusion of mean baseline scores.** All models were adjusted for age, sex, intellectual disability, presence of APOEe4, site and mean score for the respective cognitive tests included in the IICV measure. Models predicting outcomes at follow-up included time latency between assessments. Statistics reported in the table refer to the contribution of the cognitive predictor after controlling for the other variables in each model. | | | | | | |  |
| --- | --- | --- | --- | --- | --- | --- | --- |
|  | **Unstandardized**  **coefficient** | **Standardized coefficient** | **95% Confidence**  **interval for *B*** | | ***p-value*** | |  |
|  | ***B*** | ***β*** | **Lower bound** | **Upper bound** |  |  |  |
| **Dependent variable:** | | | | | |  | |
| **Baseline PET-tau Neurofibrillary Braak tangle stage**, N=136 |  |  |  |  |  |  | |
| **V-VI** |  |  |  |  |  |  | |
| *IICV- Exec* | 0.068 | 0.208 | 0.002 | 0.133 | **0.043** | |  |
| *Mean score for executive* | -0.069 | -0.245 | -0.121 | -0.006 | **0.029** | |  |
| Key: ^#^IICV denotes within-person test variability measured as the standard deviation of tests/subtest z-scores. Increased variability theoretically denotes worse cognitive function. | | | | | | |  |
|  | | | | | |  | |

| **Supplemental Table 5. Exploratory analyses of Sex x IICV**^#^ **interactions.** Linear regression models in which the interaction remained significant after inclusion of all co-variates, all involving PET tau outcomes. Models were adjusted for age, sex, the respective IICV, level of intellectual disability, presence of APOEe4 and site. For follow-up outcomes, models were also included time latency between baseline and follow-up. Statistics reported in the table refer to the contribution of the interaction term after controlling for all other variables. | | | | | | |
| --- | --- | --- | --- | --- | --- | --- |
|  | **Unstandardized**  **coefficient** | **Standardized coefficient** | **95% Confidence**  **interval for *B*** | | ***p-value*** | |
|  | ***B*** | ***β*** | **Lower bound** | **Upper bound** |  |  |
| **Dependent variable:** | | | | | |  |
| **Baseline PET-tau Neurofibrillary Braak tangle stage,** n=136 |  |  |  |  |  |  |
| **I-II** |  |  |  |  |  |  |
| *IICV- Mem x Sex (n=136)* | -0.179 | -0.738 | -0.293 | -0.065 | 0.002 | |
| **III-IV** |  |  |  |  |  |  |
| *IICV- Mem x Sex (n=136)* | -0.151 | -0.650 | -0.265 | -0.037 | 0.010 | |
| **V-VI** |  |  |  |  |  |  |
| *IICV- Mem x Sex (n=136)* | -0.199 | -0.717 | -0.341 | -0.057 | 0.007 | |
| IICV- Mem/ Exec x Sex (n=133) | -0.200 | -0.603 | -0.361 | -0.039 | 0.015 | |
| **Follow-up PET-tau Neurofibrillary Braak tangle stage**, n=78 |  |  |  |  |  |  |
| **I-II** |  |  |  |  |  |  |
| *IICV- Mem x Sex (n=79)* | -0.193 | -0.824 | -0.358 | -0.028 | 0.022 | |
| **III-IV** |  |  |  |  |  |  |
| *IICV- Mem x Sex (n=79)* | -0.279 | -1.032 | -0.468 | -0.090 | 0.004 | |
| *IICV- Exec x Sex (N=79)* | 0.251 | 0.714 | 0.027 | 0.476 | 0.029 | |
|  |  |  |  |  |  |  |
| **V-VI** |  |  |  |  |  |  |
| *IICV- Mem x Sex (n=79)* | -0.243 | -0.956 | -0.432 | -0.054 | 0.012 | |
| *IICV- Exec x Sex (n=79)* | 0.272 | 0.821 | 0.052 | 0.491 | 0.016 | |
| Key: Sex was coded as 0 = male and 1 = female. Positive interaction coefficient indicates stronger associations in females, whereas negative coefficients indicate a stronger association in males. All interactions are exploratory and were not corrected for multiple comparisons.  ^#^IICV, Intraindividual cognitive variability, denotes within-person test variability measured as the standard deviation of tests/subtest z-scores. Increased variability theoretically denotes worse cognitive function. | | | | | | |
|  | | | | | |  |
